# Prediction of Treatment Response to Neoadjuvant Chemotherapy for Breast Cancer via Early Changes in Tumor Heterogeneity Captured by DCE-MRI Registration

**DOI:** 10.1038/s41598-019-48465-x

**Published:** 2019-08-20

**Authors:** Nariman Jahani, Eric Cohen, Meng-Kang Hsieh, Susan P. Weinstein, Lauren Pantalone, Nola Hylton, David Newitt, Christos Davatzikos, Despina Kontos

**Affiliations:** 1 Department of Radiology, Perelman School of Medicine, University of Pennsylvania, Philadelphia, PA 19104 USA; 2 Department of Radiology and Biomedical Imaging, University of California San Francisco, San Francisco, CA 94115 USA

**Keywords:** Tumour biomarkers, Predictive markers, Biomedical engineering, Cancer imaging, Breast cancer

## Abstract

We analyzed DCE-MR images from 132 women with locally advanced breast cancer from the I-SPY1 trial to evaluate changes of intra-tumor heterogeneity for augmenting early prediction of pathologic complete response (pCR) and recurrence-free survival (RFS) after neoadjuvant chemotherapy (NAC). Utilizing image registration, voxel-wise changes including tumor deformations and changes in DCE-MRI kinetic features were computed to characterize heterogeneous changes within the tumor. Using five-fold cross-validation, logistic regression and Cox regression were performed to model pCR and RFS, respectively. The extracted imaging features were evaluated in augmenting established predictors, including functional tumor volume (FTV) and histopathologic and demographic factors, using the area under the curve (*AUC*) and the *C-statistic* as performance measures. The extracted voxel-wise features were also compared to analogous conventional aggregated features to evaluate the potential advantage of voxel-wise analysis. Voxel-wise features improved prediction of pCR (*AUC* = 0.78 (±0.03) vs 0.71 (±0.04), *p* < 0.05 and RFS (*C-statistic* = 0.76 ( ± 0.05), vs 0.63 ( ± 0.01)), *p* < 0.05, while models based on analogous aggregate imaging features did not show appreciable performance changes (*p* > *0*.*05*). Furthermore, all selected voxel-wise features demonstrated significant association with outcome (*p* < 0.05). Thus, precise measures of voxel-wise changes in tumor heterogeneity extracted from registered DCE-MRI scans can improve early prediction of neoadjuvant treatment outcomes in locally advanced breast cancer.

## Introduction

For women with locally advanced breast cancer, longitudinal patterns of tumor response during neoadjuvant chemotherapy (NAC) can be an important marker in evaluating treatment response and likelihood for overall survival. When dynamic contrast-enhanced magnetic resonance imaging (DCE-MRI) is part of the NAC protocol, in addition to assessing structural changes in tumor size and shape, it provides an opportunity to evaluate changes in enhancement patterns which reflect functional tumor properties as potential earlier indicators of treatment response^1–3^. Towards this end, while much progress has been made, most approaches reported to date still have important limitations by either falling short of investigating the tumor longitudinally or by overlooking the finer details of the longitudinal imaging phenotype by primarily relying on aggregate measures of tumor structure and function^4–7^. For example, although Hylton *et al*.^6^ have shown that measuring the aggregate change of functional tumor volume (FTV) during NAC can be an indicator of pathologic complete response (pCR) and long-term recurrence-free survival (RFS), FTV does not adequately capture intra-tumor heterogeneity which has increasingly been shown to be a major indicator of tumor aggressiveness and treatment resistance^8^.

As tumors are known to be temporally and spatially heterogeneous and tend to deform regionally during treatment^9,10^, more precise and longitudinal quantification of phenotypic tumor heterogeneity could provide new insight for early prediction of treatment response and long-term survival. To calculate regional longitudinal changes, deformable image registration techniques can be used to match images from different imaging sessions voxel-by-voxel^11,12^. However, the lack of robust image registration techniques has led to many breast cancer investigations to overlook voxel-wise approaches for capturing such heterogeneous tumor changes^12,13^. Recently, a registration method based on attribute-matching^14^ has been developed shown to have improved accuracy compared to conventional intensity-based registration methods^15,16^. Implementing an accurate image registration technique, a parametric response map (PRM)^17,18^, as well as regional deformation measures^19–21^, can provide quantitative voxel-based information regarding heterogeneous changes within the tumor during treatment.

We evaluated phenotypic changes in tumor heterogeneity, quantified with voxel-wise image registration, for augmenting early prediction of pCR and RFS after NAC for locally advanced breast cancer. The rationale is to benefit from early-treatment information, captured via a robust deformable image registration technique, in order to precisely quantify voxel-wise changes in morphologic, structural, and kinetic tumor features. We hypothesize that imaging markers capturing such early changes within the tumor can improve prediction of pCR and RFS for women diagnosed with locally advanced breast cancer, and thus providing additional information to help better guide their treatment.

## Methods

### Patient population and data acquisition

This study was approved by the institutional review board of University of Pennsylvania. No consent or waiver was required as data were obtained de-identified from the National Cancer Institute’s Cancer Imaging Archive^22^. The patient population analyzed for our study was a subset of the multicenter Investigation of Serial Studies to Predict Your Therapeutic Response with Imaging and moLecular Analysis and American College of Radiology Imaging Network 6657 trial (I-SPY 1 TRIAL/ACRIN 6657) which recruited women with T3 tumors who received anthracycline-cyclophosphamide NAC^2^. Four MR imaging examinations were performed, including pre-treatment (first examination four weeks before the treatment), early-treatment (second examination performed at least two weeks after the first cycle of chemotherapy) and between treatments (third examination), and fourth examination performed before surgery and after completion of NAC.

The data acquisition was previously described according to the ACRIN 6657/ISPY-1 protocols^2^. In summary, DCE-MR scans were collected from nine different centres using 1.5-T MR imaging systems with a dedicated breast radiofrequency coil was used to acquire pre- and post-contrast images at each examination. The imaging procedure included a localization scan and T2-weighted sequences followed by T1-weighted of DCE-MRI series. The T1-weighted sequence was acquired once before contrast injection and at least twice afterwards. The first two contrast-enhanced images were acquired 2.5 and 7.5 minutes after contrast injections.

Clinical, demographic, and histopathologic data including age, race and hormone receptor status (coded as a three-level categorical variable: 1. HR-positive and Her2-negative, 2. Her2-positive, 3. triple negative) were available for each patient (Table 1), as well as functional tumor volume measurements at pre-treatment (*FTV*_*1*_) and early-treatment visits (*FTV*_*2*_). Furthermore, the RFS outcomes were reported and measured according to the STEEP criteria^23^, as the time from the first cycle of chemotherapy to disease recurrence or death. The pCR outcomes were also defined as no remaining invasive cancer in axillary lymph nodes or breast^24^.

**Table 1:** Patient characteristics for the ISPY-1/ACRIN 6657 trial sample analyzed in our study.

Age	
Mean (sd)	47.87 (8.9)
Race	
White or Hispanic	98 (74.2%)
Other	34 (25.8%)
Bilateral cancer	
No	130 (98.4%)
Yes	2 (1.6%)
Laterality	
Left	65 (49.2%)
Right	67 (50.8%)
Estrogen receptor (ER) status	
Negative	62 (46.9%)
Positive	70 (53.1%)
Progesterone receptor (PR) status	
Negative	74 (56%)
Positive	58 (44%)
HER2 status	
Negative	83 (62.9%)
Positive	49 (37.1%)
Hormone receptor status	
HER2	49 (37.1%)
HR+ and HER2−	51 (38.6%)
triple negative	32 (24.3%)
RFS	
Censor	93 (70.5%)
Event (recurrence or death)	39 (29.5%)
pCR	
No	89 (67.4%)
Yes	38 (28.8%)
Missing	5 (3.8%)

**Figure 1:**
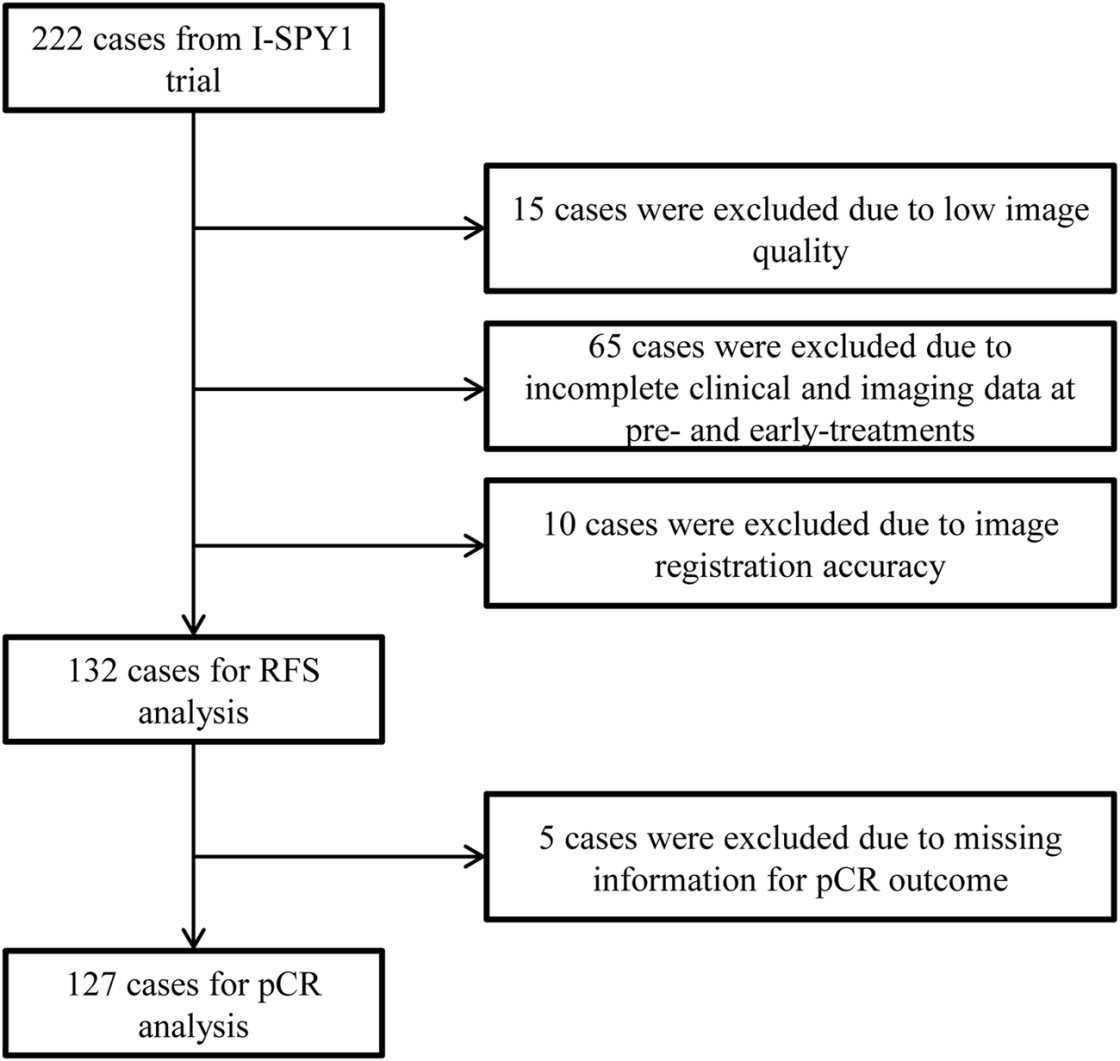
Inclusion and exclusion criteria from the ISPY-1/ACRIN 6657 trial cohort.

For our study, we focused only on the information extracted from the first two MRI examinations (i.e., at pre-treatment and early-treatment visits), as outcome prediction before the initiation or early in the course of treatment would be of particular clinical value. From the available 222 I-SPY 1/ACRIN 6657 trial participants, limiting ourselves to those with complete clinical and imaging data at pre-treatment and early-treatment visits reduced the analysis set to 142 patients; excluding an additional 10 for whom image registration could not complete (see the next section for details of registration). That resulted in a sample of 132 patients available for RFS analysis in our study. pCR information was missing for 5 participants, leaving 127 patients for pCR analysis (Fig. 1).

### Image pre-processing

Three image pre-processing steps were implemented before quantitative analyses. First, a nonparametric non-uniform intensity normalization (N3) method was implemented for bias-field correction to reduce the negative impact of MR imaging artifacts. Then, histogram matching was done between images at pre- and early-treatment for more accurate registration implementation. Finally, all images from different patients were resampled to the same spatial resolution to have consistent intensity values for feature extraction.

### Deformable image registration and tumor segmentation

**Figure 2:**
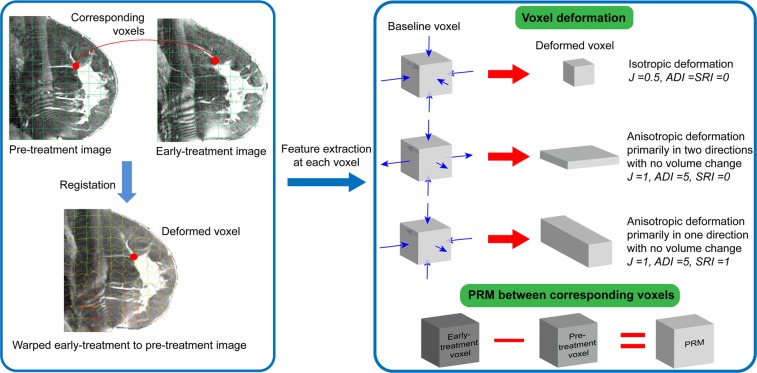
Deriving the deformation field from image registration (left) to extract longitudinal voxel-wise image features (right).

After image pre-processing steps, we applied a deformable image registration algorithm to spatially and anatomically align the early-treatment MR images to the pre-treatment ones^14^ (Fig. 2). The registration algorithm is based on attribute matching and mutual-saliency weighting. This registration calculates a spatial transformation, *T*, mapping each voxel, **x**, to its image, *T*(*x*). *T* is computed by minimizing a cost function, *E*, that is a function of (1) mutual saliency, where *ms*(*x*_1_, *x*_2_) measures the dissimilarity between two voxels; and (2) attribute matching, where *A*(*x*) is a vector encoding the anatomic and geometric properties of a voxel:

1 $$E=\mathop{\int }\limits_{\begin{array}{c}x{\epsilon }\\ breast\\ volume\end{array}}ms(x,T(x))(\frac{1}{d})\Vert {A}_{1}(x)-{A}_{2}{(T(x))\Vert }^{2}dx$$

where *d* is the number of image dimensions.

The algorithm has been previously validated for longitudinal MR image registration for breast cancer and shown to be significantly more accurate compared to conventional intensity-based registration algorithms^19^. The rationale in our study is that this accurate matching allows for monitoring changes within the corresponding voxels between pre- and early-treatment images. Registration was, therefore, first applied to the entire breast, and then, subsequent voxel-wise image analyses were performed within the tumor region of the pre-treatment image (*FTV*_*1*_). Thus, *FTV*_*1*_ mask was applied to both pre-treatment and registered early-treatment images to track voxel-wise changes within the initial tumor region. A signal enhancement ratio method was used to analyze DCE-MR images and segment functional tumor volumes (*FTV*_*1*_ and *FTV*_*2*_)^6,25^.

### Voxel-wise longitudinal imaging features

#### Feature maps extraction

Comparing each pair of corresponding voxels extracted from the registration of pre- and early-treatment DCE-MRI scans, two groups of imaging features were computed to quantify tumor changes: (i) voxel-wise tumor deformation, and (ii) voxel-wise changes of kinetic features (PRM of kinetic features):

Voxel deformation measures: Evaluation of voxel deformation provides the opportunity to track how the tumor deforms in response to therapy by quantifying regional changes in tumor size, shape and orientation. Specifically, three independent voxel-wise measures of tumor deformation were calculated: (1) *Jacobian*, representing the volume expansion or contraction of each voxel computed as the ratio of the volume at early-treatment image to the corresponding volume at pre-treatment image in a given point, (2) the anisotropic deformation index (*ADI*) a measure of the magnitude of the anisotropic (non-shape-preserving) deformation at each voxel, and (3) the slab-rod index (*SRI*) a measure of the shape (orientation preference) of the anisotropic deformation (Fig. 2):

The transformation function *T* derived from image registration extracts information regarding voxel-wise changes in volume and shape between pre-treatment and early-treatment imaging. At a given voxel, the eigenvalues of ∇*T*(∇*T*)^*T*^, λ_1_, λ_2_, and λ_3_, denote the principal strains where λ_1_ > λ_2_ > λ_3_.

#### Jacobian

The Jacobian, *J*, is the voxel-wise volume ratio between early-treatment and pre-treatment images, indicating local contraction (*Jacobian* < *1*) or local expansion (*Jacobian* > *1*).

2 $$Jacobian=\frac{{v}_{early-treatment}}{{v}_{pre-treatment}}={\lambda }_{1}{\lambda }_{2}{\lambda }_{3}$$

#### Anisotropy indices

However, the Jacobian is unable to capture information about the directionality and shape of local deformation. The anisotropic deformation index (*ADI*) and the slab-rod index (*SRI*)^26^ capture two such measures.

The *ADI* defined at each point as

3 $$ADI=\sqrt{{(\frac{{\lambda }_{1}-{\lambda }_{2}}{{\lambda }_{2}})}^{2}+{(\frac{{\lambda }_{2}-{\lambda }_{3}}{{\lambda }_{3}})}^{2}}$$

It measures how much the local transformation is anisotropic (directional). The ADI ranges from 0 to ∞; when λ_1_ = λ_2_ = λ_3_, the ADI is zero implying isotropic deformation (deformation that is equal in all directions, shape-preserving deformation), and larger ADI indicates more anisotropy (Fig. 2).

The SRI defined at each point as

4 $$SRI=\frac{ta{n}^{-1}({\lambda }_{3}({\lambda }_{1}-{\lambda }_{2})/{\lambda }_{2}({\lambda }_{2}-{\lambda }_{3}))}{\pi /2}$$

shows whether the voxel deforms mainly in one direction (rod-like deformation, *SRI* ≈ 1) or two directions (slab-like, *SRI* ≈ 0) (Fig. 2).

PRMs of kinetic features: Besides deformation, image registration allows for constructing voxel-wise maps of changes in enhancement patterns extracted from kinetic features in DCE-MRI, which can be a means of characterizing intra-tumor functional heterogeneity^27^. Here, we hypothesize that such voxel-wise measures can also quantify *de novo* changes in tumor heterogeneity which can be early indicators of therapy resistance, and thus markers of treatment response.

During the acquisition of DCE-MRI scans (a pre-contrast image, at time point *t*_*0*_, followed by two post-contrast images, taken at two different delay times after injection of the contrast agent, *t*_*1*_ and *t*_*2*_, respectively), signal intensity of each voxel can be recorded at each time point (*I*(*t*)). From that, four kinetic features were computed to quantify the enhancement pattern for each voxel: peak enhancement (*PE*), wash-in slope (*WIS*), wash-out slope (*WOS*), and signal enhancement ratio (*SER*).

5 $$PE=\mathop{{\rm{\max }}}\limits_{t={t}_{PE}}\frac{I(t)-I({t}_{0})}{I({t}_{0})}$$

6 $$WIS=\{\begin{array}{cc}\frac{PE}{{t}_{PE}-{t}_{0}} & if\,{t}_{PE}\ne 0\\ 0 & otherwise\end{array}$$

7 $$WOS=\{\begin{array}{cc}\frac{I({t}_{2})-I({t}_{1})}{{t}_{2}-{t}_{PE}} & if\,{t}_{2}\ne {t}_{PE}\\ 0 & otherwise\end{array}$$

8 $$SER=\frac{I({t}_{2})-I({t}_{0})}{I({t}_{1})-I({t}_{0})}$$

For a given kinetic feature *F*, to analyze the voxel-wise change in *F* between the pre-treatment and early-treatment visits, we constructed the parametric response map (*PRM*) for *F*. Given the transformation *T* between pre-treatment voxels and their corresponding voxels in the early-treatment image, the PRM (of *F*) at any voxel **x** is defined as

9 $$PRM(x)=J\times {F}_{early-treatment}(T(x))-{F}_{pre-treatment}(x)$$

*J* here is the Jacobian, the proportional volume change at **x** between visits, which scales the value in cases when a voxel in one image corresponds to a larger or smaller volume in the other image.

#### Heterogeneity indices of the imaging features

Based on prior research^17,18^, within the *FTV*_*1*_ of each tumor, we calculated feature values as the fraction of voxels for each corresponding *Jacobian* and PRM of kinetic features whose value increased between pre- and early treatment visit (i.e., number of voxels with positive value/total number of voxels).

For the *ADI* and *SRI* features, since anisotropic deformation indicates a single relative measure between each corresponding voxels, we calculated the entropy of the corresponding *ADI* and *SRI* feature maps to specifically quantify the heterogeneity of the tumor deformation^28^.

10 $$Entropy=\mathop{\sum }\limits_{i=1}^{N}\,P(i)lo{g}_{2}P(i)$$

where *N* is the number of values the measure takes over all voxels in the tumor, and *P*(*i*) is the probability that the feature will be equal to level *i* at any given voxel. (When *ADI* or *SRI* is equally likely to take every value that it takes over the image, entropy is low; when it takes some values frequently and others infrequently, entropy is high).

**Figure 3:**
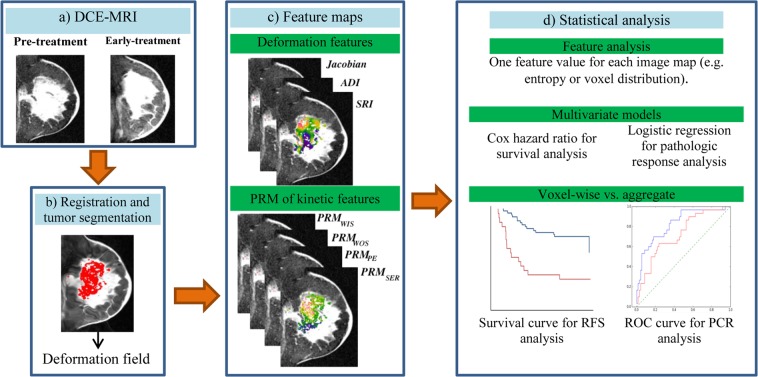
Outline of the process of voxel-wise analysis using deformable registration. (**a**) Acquiring DCE-MRI before and during neoadjuvant chemotherapy. (**b**) Applying deformable image registration to derive the transformation field, followed by segmentation of functional tumor volume. (**c**) Utilizing the deformation field for voxel-wise quantification of two groups of feature maps within the tumor: 1. Voxel-wise deformation features, including the Jacobian showing regional volume ratio and anisotropic deformation measures of directional deformation 2. Parametric response maps (PRM) showing kinetic feature variation. (**d**) Building multivariable models using the top voxel-wise feature values, and comparing their performance with that of the corresponding aggregate feature models in predicting recurrence-free survival (RFS) and pathologic complete response (pCR).

These computations resulted in a total of seven measures for each tumor, namely the proportion of increasing voxels for each of the *Jacobian*, *PE*, *SER*, *WIS*, and *WOS* features, and the entropy of the *ADI* and *SRI* measures (Fig. 3).

### Analogous aggregate longitudinal features

To compare the performance of the proposed voxel-wise imaging features with currently established DCE-MRI measures, we calculated analogous, longitudinal tumor-wide aggregate features (i.e., mean values within FTVs). As an aggregate analogue to the *Jacobian*, we calculated the tumor-wide change in the entire volume (*FTV*_*2*_*/FTV*_*1*_). For each kinetic feature, the corresponding aggregate feature was calculated as the change in its average value:

11 $${{\rm{\Delta }}}_{f}=({f}_{early-treatment}-{f}_{pre-treatment})/{f}_{early-treatment}$$

where *f* is the average value of the feature (*PE*, *WIS*, *WOS*, *SER*) over the whole tumor. Aggregate features for the pre-treatment and early-treatment images were calculated over *FTV*_*1*_ and *FTV*_*2*_, respectively. This resulted in five imaging features for aggregate analyses. No corresponding aggregate features were calculated for *ADI* and *SRI*, as these measure voxel-wise orientational changes captured specifically and only by image registration.

### Statistical analysis

First, a baseline model including the established covariates of age, race, hormone receptor status, and tumor volume (in this case, *FTV*_*2*_) was built and tested. Then, features extracted from both voxel-wise and aggregate measures were tested as additions to this baseline model. Logistic regression was performed to assess the strength of associations of features with pCR, where the area under the receiver-operating-characteristic curve (AUC) was used to assess model performance. Cox proportional hazard modeling was used for time-to-event analysis to assess the strength of association of features with RFS, where the C-statistic was used as a measure of predictive performance^29^.

For both pCR and RFS, five-fold cross-validation was performed, where the best model for each cross-validation loop was selected in two steps: first, using only the training set, each of the seven voxel-wise imaging features (five imaging features for the aggregate analysis) was evaluated as a univariable addition to the baseline model, and features were ranked based on their performance (AUC for pCR and C-statistic for RFS). Then, best subset model selection was used where seven (again, five for aggregate models) models were built and evaluated: one with the single best feature, one with the two best features, and so on, where the Akaike information criterion (AIC) was used to choose the best multivariable model from these seven (or five) models. Finally, the selected model was applied to the unseen test set, and the AUC or C-statistic was calculated. Averaging over all five cross-validation loops, the mean AUC or C-statistic was used as the final, cross-validated, measure of model performance.

To estimate the odds ratios or hazard ratios for each model (voxel-wise or aggregate), the features selected in more than 80% of the cross-validation loops (4 or 5) were then used in multivariable models fitted to the full dataset. Using the likelihood ratio test, the proposed voxel-wise and aggregate models were compared with the baseline model to assess their added value as covariates. Furthermore, RFS analysis was evaluated via Kaplan-Meier plots and survival ratios derived from hazard as predicted from the model — a participant’s *risk signature* — dichotomized at the median into high- and low-risk groups. For a given model, the risk signature of each participant was defined as that participant’s values of the covariates in the model (age, race, hormone receptor status, FTV_2_, and selected imaging features) weighted by the corresponding coefficients of those covariates in the model, to arrive at a predicted risk score^30,31^. The *p-value* of 0.05 cutoff was used to determine statistical significance throughout. Statistical analysis was conducted using R (R version 3.3.2, R Foundation for Statistical Computing, Vienna, Austria).

## Results

### Patient population

Of 132 participants used in our study for RFS analysis, 39 had an event (recurrence or death), over a median follow-up time of 3.62 years. Of the 127 participants for whom the pCR outcome was recorded, 38 experienced pCR (Table 1).

### Pathologic complete response

The baseline model for pCR had a mean cross-validated AUC = 0.71 (Supporting Information Table S1). Hormone receptor status had a statistically significant association with pCR in this multivariable model (odds ratio: 2.06, *p* < 0.05) whereas *FTV*_*2*_ and other clinical variables had no statistically significant associations with treatment response (*p* > 0.05). Adding the voxel-wise features to the baseline features and using the best models derived as described above (Supplementary Table S2), the performance of the baseline model was improved significantly (*p* < 0.05), resulting in mean cross-validated AUC of 0.78 (Table E3). The voxel deformation features (*Jacobian*, *ADI* and *SRI*) were selected in all five folds, while the PRM features, *PRM*_*WOS*_ and *PRM*_*PE*_ were selected twice and once, respectively (Supplementary Table S3 for selected features in each fold). In the aggregate-measures models, although some features showed consistency in being selected among training sets (e.g., *FTV*_*2*_/*FTV*_*1*_ and *∆*_*WIS*_ were selected in four out of five folds), no model demonstrated improvement in performance (AUC = 0.71, *p* > 0.05). A model based on only the voxel-wise features showed mean cross-validated AUC of 0.74, demonstrating better performance than the baseline and aggregate models, despite not incorporating standard baseline covariates such as *FTV*_*2*_ and hormone receptor status. Fitting the multivariable model to the full dataset, all three selected voxel-wise imaging features showed statistically significant associations with pCR (*p* < 0.05), while *FTV*_*2*_ and other aggregate features had no statistically significant associations with pCR (Table 2). Furthermore, the model augmented with voxel-wise features showed a statistically significant improvement over the baseline model, as determined by the likelihood ratio test (*p* < 0.001) while the proposed features in the aggregate model did not (*p* = 0.14).

**Table 2:** Statistical analysis of voxel-wise versus aggregate features in multivariable pCR models.

	Voxel-wise analysis			Aggregate analysis		
	Selected Features	Odds ratio (95% CI)	p-value	Selected Features	Odds ratio (95% CI)	p-value
Clinical features	*Age*	0.97 (0.93–1.02)	0.241	*Age*	0.81 (0.53–1.25)	0.351
	*Race*	1.73 (0.61–4.87)	0.307	*Race*	1.14 (0.74–1.74)	0.524
	*Hormone receptor status*	2.13 (1.21–3.90)	0.010^†^	*Hormone receptor status*	2.04 (1.21–3.59)	0.009†
Tumor volume	*FTV*_2_	0.53 (0.23–0.95)	0.075	*FTV*_2_	0.65 (0.32–1.07)	0.158
Proposed features	*Jacobian*	0.30 (0.12–0.62)	0.003^†^	*FTV*_2_/*FTV*_1_	0.74 (0.41–1.19)	0.263
	*ADI*	1.89 (1.10–3.41)	0.025^†^	Δ_*WIS*_	1.30 (0.87–2.30)	0.248
	*SRI*	1.92 (1.00–3.98)	0.041^†^			

^†^*p *< 0.05.

### Recurrence-free survival

The baseline model gave a mean cross-validated C-statistic of 0.63 (Supplementary Table S1). Fitting this model to the full dataset showed a statistically significant association of *FTV*_*2*_ with RFS (hazard ratio: 1.81, *p* < 0.001) while age, race, and hormone receptor status did not show associations with RFS (Supplementary Table S1). When adding the voxel-wise features to the baseline model (Supplementary Table S4), *PRM*_*PE*_ and *PRM*_*WIS*_ were selected in all five folds, and Jacobian and *SRI* were selected in 4 folds (Supplementary Table S5). Voxel-wise models performed significantly better than the baseline model (*p* < 0.05), with a mean cross-validated C-statistic of 0.76 (Supplementary Table S5). Furthermore, a model based on only the voxel-wise features gave a mean cross-validated C-statistic of 0.73, showing better performance than the baseline model even without predictors such as *FTV*_*2*_ and hormone receptor status. The aggregate-feature models had even lower performance with a mean cross-validated C-statistic of 0.61 (Supplementary Table S5).

Building a Cox model on the full dataset, including the baseline features, and the selected voxel-wise features *PRM*_*PE*_, *PRM*_*WIS*_, Jacobian, and *SRI* (these features were included in four or five of the cross-validation runs), all the voxel-wise features showed statistically significant associations with RFS (Table 3). In contrast, in an analogous model, none of the aggregate imaging features had a statistically significant association with RFS (Table 3). As was true for pCR modeling, the likelihood ratio test showed that augmenting the model with voxel-wise features resulted in a statistically significant improvement over the baseline model, (*p* < 0.001) while adding the aggregate features did not (*p* = 0.23).

**Table 3:** Statistical analysis of voxel-wise versus aggregate features in multivariable RFS models.

	Voxel-wise analysis			Aggregate analysis		
	Selected Features	Hazard ratio (95% CI)	p-value	Selected Features	Hazard ratio (95% CI)	p-value
Clinical features	*Age*	0.90 (0.63–1.28)	0.588	*Age*	0.93 (0.64–1.35)	0.810
	*Race*	1.04 (0.74–1.48)	0.784	*Race*	0.96 (0.67–1.37)	0.656
	*Hormone receptor status*	1.05 (0.72–1.53)	0.786	*Hormone receptor status*	0.96 (0.66–1.38)	0.714
Tumor volume	*FTV*_*2*_	1.72 (1.28–2.31)	0.002^†^	*FTV*_*2*_	1.95 (1.42–2.69)	<0.001^†^
Proposed features	*Jacobian*	0.59 (0.38–0.93)	0.023^†^	*FTV*_2_/*FTV*_1_	0.91 (0.61–1.36)	0.664
	*PRM*_*PE*_	2.66 (1.65–4.27)	<0.001^†^	Δ_*PE*_	0.90 (0.57–1.44)	0.686
	*PRM*_*WIS*_	0.56 (0.35–0.89)	0.016^†^	Δ_*WOS*_	1.33 (0.97–1.81)	0.067
	*SRI*	2.07 (1.02–4.18)	0.041^†^			

^†^*p* < 0.05.

**Figure 4:**
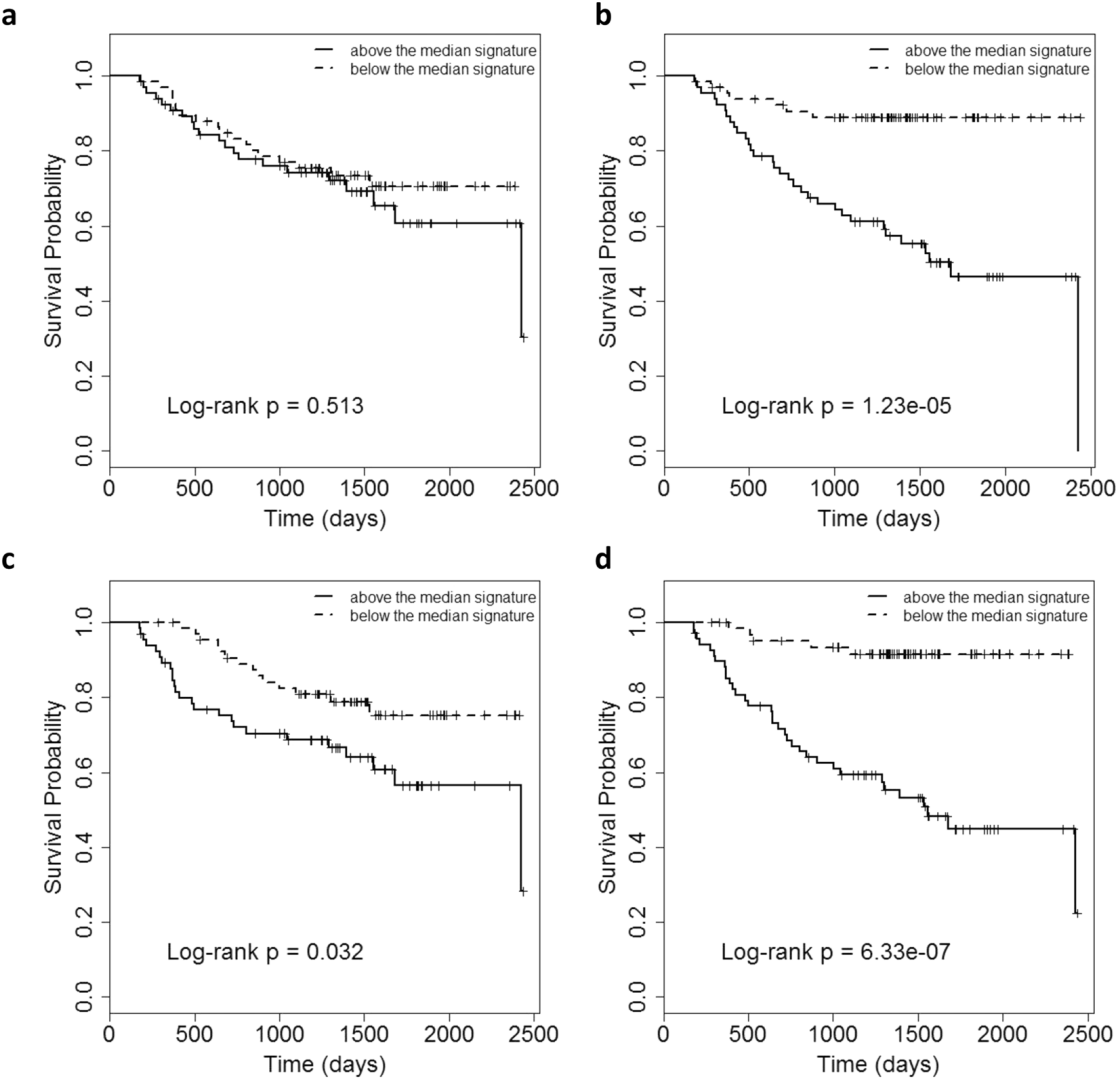
Kaplan Meier plots of RFS in cross-validated datasets showing the performance of selected (**a**) aggregate, (**b**) voxel-wise and (**c**) baseline and (**d**) baseline plus voxel-wise features. The voxel-wise model indicated a high performance (log-rank *p* < 0.001), while the aggregate features were unable to separate high-risk from low-risk patients (log-rank *p* = 0.513). Also, the selected voxel-wise features in combination with the baseline features could improve the baseline model significantly (*p* < 0.05).

Finally, splitting the patients into low and high-risk groups based on their median risk score signature (Fig. 4) gave a significantly higher ratio (i.e., greater separation) of survival probabilities between high-risk and low-risk patients, when modelling was performed via the final multivariable voxel-wise imaging signature (log-rank *p* < 0.001) rather than using the corresponding selected aggregate features (log-rank *p* = 0.51). Furthermore, when combining the voxel-wise features with the baseline predictors, the performance improved significantly (ratio at median survival time = 1.55, log-rank p < 0.001) compared to the performance of the baseline predictors alone (ratio at median survival time = 1.11, log-rank *p* = 0.032).

### Voxel-wise versus aggregate representations

**Figure 5:**
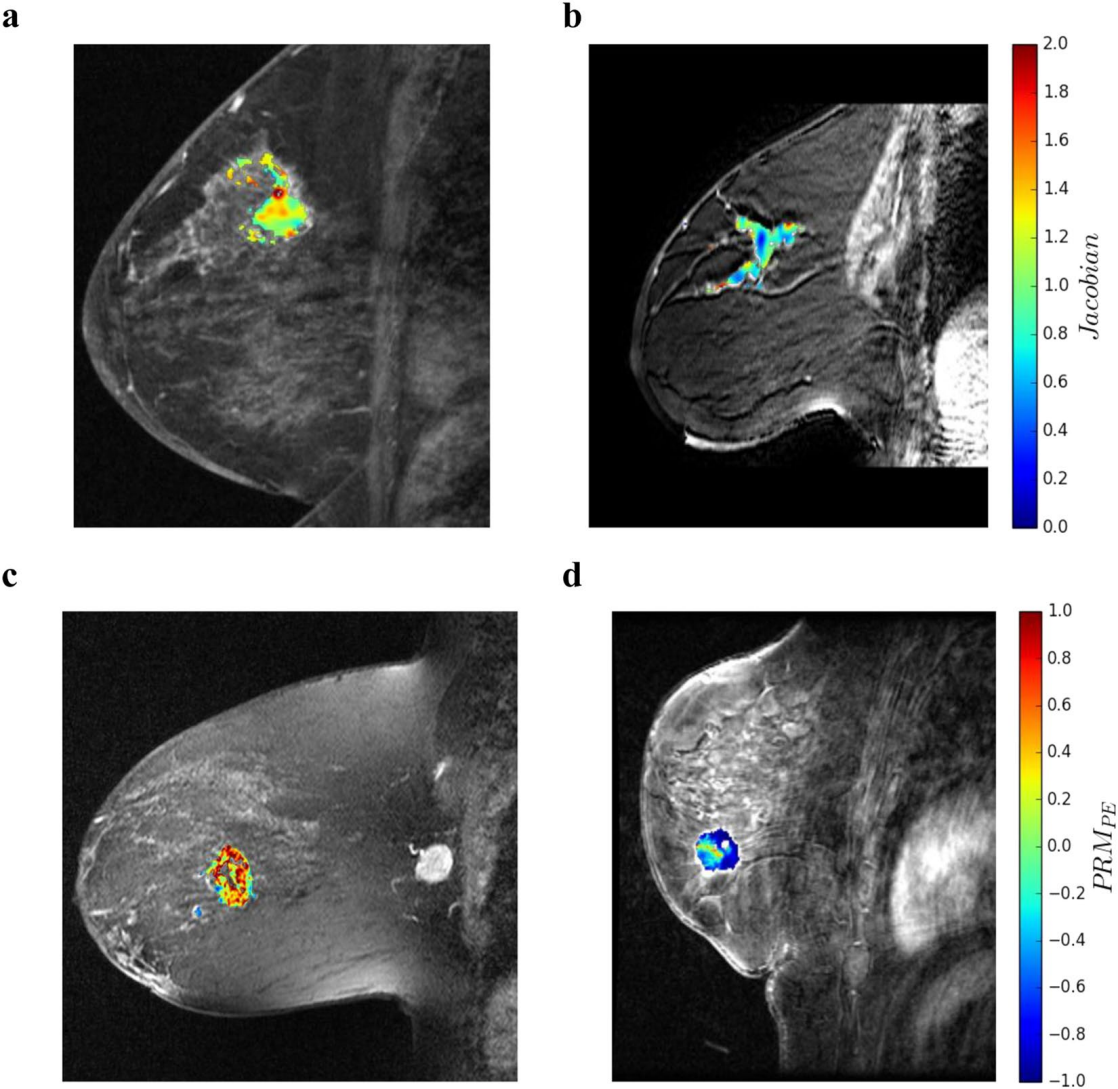
Voxel-wise feature map distributions within the tumor. (**a**,**b**) Distribution of Jacobian within FTV_1_ for two representative patients (left: age 59, triple negative, no event; right: age 62, Her2-positive and a future recurrence) with similar volume change ratio (FTV_2_/FTV_1_ ≈ 0.85) for both patients. However, 64% of tumor voxels in the patient with no recurrence showed expansion (Jacobian >1) while only 25% showed expansion for the patient with recurrence. (**c**,**d**) Distribution of PRM_PE_ within FTV_1_ for two representative patients (left: age 39, HR-positive and Her2-negative, future recurrence; right: age 58, HR-positive and Her2-negative, no event) with similar *PE*_*2-mean*_ − *PE*_*1-mean*_ ≈ 0.50 for both patients. However, 67% of tumor voxels showed increased PE in the patient with future recurrence versus only 20% in the patient without an event.

To better understand the differences between voxel-wise and aggregate representations, Fig. 5 demonstrates feature maps for a few representative patients, showing how patients with similar aggregate feature values may have different heterogeneity due to different voxel-wise distributions. Supplementary Figs S1 and S2 show the distributions of proposed imaging features according to the treatment outcomes. All feature values except the entropy of *ADI* for pCR analysis indicated distinct distribution (*p* < 0.05). Furthermore, Supplementary Table S6 summarizes how to interpret the proposed voxel-wise imaging features to improve the prediction of pCR and RFS.

## Discussion

The importance of early-treatment response assessment in optimizing patient care and treatment adjustment have been proven^32,33^. Our study suggests that voxel-wise longitudinal analyses of DCE-MR images can quantify heterogeneous changes within the tumor as an indicator of therapy response and improve prediction of RFS and pCR, compared to conventional tumor volume and aggregate kinetic measures, as early as the first treatment time point in NAC. Importantly, the proposed voxel-wise features provide information independent of conventional predictive covariates such as age, race, hormone receptor status, and tumor volume.

Using registration, we extracted two types of feature maps from the longitudinal data: voxel-wise deformation, and PRMs of kinetic features. The anisotropy indices (*ADI* and *SRI*) in combination with the Jacobian, provide a complete descriptor of local tumor deformation^26^, which can capture heterogeneous changes within tumor transformations^34^. Features based on the PRMs of kinetic features are also important in capturing functional tumor heterogeneity regarding changes in enchantment patterns to augment models of pCR and RFS. It should be noted that the consistent selection of the voxel-wise features in most training folds (80% of folds) of the cross-validation suggests that they were robust across training sets. The combination of techniques in our study —robust registration; use of voxel-wise measures; use of deformation measures and PRMs of kinetic features — provide statistically significant improvements over previous similar analyses with conventional tumor volume measures and aggregate kinetic features in predicting RFS and pCR^2,6^.

Although recent investigations for pCR prediction attempted to characterize tumor heterogeneity during chemotherapy, quantification of heterogeneity was performed separately at different time points^35^ without the incorporation of image registration, and relative changes were measured by averaging corresponding feature values over time^13^. Cho *et al*. evaluated the PRM of signal intensity during chemotherapy to predict pCR but in sub-volumes rather than voxels^36^. Our results suggest that using longitudinal voxel-wise markers, even without tumor volume, can outperform conventional approaches for the prediction of both RFS and pCR.

There were some limitaions in our study, one was the relatively small sample size of the patients (132 participants for RFS, 127 for pCR) with a low number of events (39 for RFS and 38 for pCR). We, therefore, limited our evaluation to a single first-order feature value for each type of our measures (i.e., percent of voxels with relative increase between pre- and early- treatment scans and entropy of anisotropic deformations) to avoid overfitting and used five-fold cross-validation to get a preliminary estimate of the generalizability of our findings. In addition, although we showed significant improvement in predicting pCR and RFS by extracting voxel-wise temporal feature changes, when the I-SPY 1 TRIAL was conducted (i.e., from May 2002 to March 2006) temporal resolutions were set to 2.5 and 7.5 minutes for post-contrast images. As DCE-MRI was still relatively in its early stages, these temporal resolutions were considered standard of care, especially when considering the multi-institutional setting of I-SPY 1 and the need to standardize acquisitions across sites. However, recent advances in MRI techniques provide significantly higher temporal resolution, and according to recommendations from EUSOMA for breast imaging, the minimum temporal resolution should be less than 2 minutes^37^. It has been shown that the most informative feature values for tumor characterization should be available at 2 minutes or less after the injection of contrast agent^38^. Thus, the delayed phase of post-contrast images in this study may not fully utilize the most valuable feature information in predicting pCR and RFS. We hypothesize that using the proposed feature maps with more current, advanced MRI techniques would enhance the prediction of RFS and pCR even further.

Furthermore, since neoadjuvant trastuzumab was not used as standard therapy until 2005, most patients with HER2+ were only under neoadjuvant chemotherapy in this study; there were only a few (n = 16) that got trastuzumab but those were excluded from the original I-SPY 1 trial imaging analysis for consistency^6^, which we also did for the purposes of our study. However, currently, patients with HER2+ usually also use targeted therapy drugs including trastuzumab (Herceptin) and pertuzumab (Perjeta) which improve pathologic complete response and overall survival when added to chemotherapy^39^. Therefore, it would be necessary to investigate the performance of the imaging signatures proposed here on the outcomes for HER2+ patients who have received the targeted therapies in addition to neoadjuvant chemotherapy.

To address above limitations, we plan to perform such an evaluation when the imaging data from a larger independent validation data set become available. The current work can also be extended by applying these analyses to longitudinal images at additional mid- and late- treatment time points to better characterize heterogeneous tumor responses and the effects of treatment over time. Also, combining these first-order voxel-wise deformations and PRMs of kinetic features with second- and third order voxel-wise imaging features, such as texture and shape-based features, may provide even more predictive signatures in treatment response assessment^30,40^.

In conclusion, we demonstrated that evaluation of voxel-wise changes in longitudinal analyses of DCE-MR images can reveal valuable phenotypic tumor heterogeneity markers to significantly improve early therapy response prediction compared to conventional tumor volume and aggregate kinetic measures, as early as the first treatment time point. Such phenotypic markers can be derived from imaging that is the current standard of care in neoadjuvant chemotherapy response assessment, and thus potentially provide valuable information with no additional invasive procedures to better tailor treatment selection for individual patients.

## Supplementary information

Supplementary Information_sr
