# Supplementary material for: Prediction of Treatment Response to Neoadjuvant Chemotherapy for Breast Cancer via Early Changes in Tumor Heterogeneity Captured by DCE-MRI Registration
Source: Sci Rep. 2019 Aug 20;9:12114. doi: 10.1038/s41598-019-48465-x (PMC6702160; doi:10.1038/s41598-019-48465-x)
Supplement: Supplementary file 1 — Supplementary Information_sr [file 41598_2019_48465_MOESM1_ESM.docx]

Prediction of Treatment Response to Neoadjuvant Chemotherapy for Breast Cancer

via Early Changes in Tumor Heterogeneity Captured by DCE-MRI Registration

Nariman Jahani, PhD^1^, Eric Cohen, MS^1^, Meng-Kang Hsieh, MS^1^, Susan P. Weinstein, MD^1^, Lauren Pantalone, BS^1^, Nola Hylton, PhD^2^, David Newitt, PhD^2^, Christos Davatzikos, PhD^1^, Despina Kontos, PhD^1*^

^1^ Department of Radiology, Perelman School of Medicine, University of Pennsylvania, Philadelphia, PA, 19104

^2^ Department of Radiology and Biomedical Imaging, University of California San Francisco, San Francisco, CA, 94115

Supplementary Information

Table S 1 Multivariable analyses of the baseline model using (a) the full dataset for hazard ratio and odds ratio assessment; (b) the mean of their performances as tested on each five-fold cross-validation set.

| **(a)** | **pCR** | | **RFS** | |
| --- | --- | --- | --- | --- |
| **Feature** | Odds ratio (95% CI) | p-value | Hazard ratio (95% CI) | p-value |
| *Age* | 0.86(0.56-1.30) | 0.479 | 0.99(0.95-1.03) | 0.640 |
| *Race* | 1.19(0.79-1.79) | 0.386 | 0.93(0.42-2.04) | 0.850 |
| *Hormone receptor status* | 2.06(1.22-5.57) | 0.007^†^ | 1.06(0.67-1.66) | 0.818 |
| *FTV_2_* | 0.57(0.29-0.96) | 0.069 | 1.81(1.35-2.42) | <0.001^†^ |
| **(b)** | **pCR** | | **RFS** | |
| Mean cross-validated AUC (SE) | 0.71(0.04) | | - | |
| Mean cross-validated C-statistic (SE) | - | | 0.63 (0.01) | |

† p <0.05

Table S 2 An example of choosing the best model on a particular fold (fifth fold in this case), from among the best-subset models for 1–7 voxel-wise or 1-5 aggregate features. All features were evaluated as additions to the baseline features. The full model-building procedure does this within each of the five folds.

| **Number of added features** | **Voxel-wise features** | | **Aggregate features** | |
| --- | --- | --- | --- | --- |
|  | Cross-validated AUC (95% CI) | AIC* | Cross-validated AUC (95% CI) | AIC* |
| 1 | 0.79(0.69-0.89) | 111 | 0.77(0.66-0.87) | 112 |
| 2 | 0.81(0.72-0.91) | 106 | 0.77(0.67-0.88) | 113 |
| 3^±^ | 0.83(0.74-0.92) | 105 | **0.77(0.67-0.88)** | **112** |
| 4^†^ | **0.85(0.77-0.94)** | **102** | 0.78(0.67-0.88) | 114 |
| 5 | 0.86(0.77-0.94) | 104 | 0.78(0.68-0.88) | 118 |
| 6 | 0.86(0.78-0.94) | 106 | -- | -- |
| 7 | 0.86(0.78-0.94) | 108 | -- | -- |

* Lower AIC indicates better model, ± Selected as the best aggregate model in the fifth fold. This particular model used the features FTV_2_/FTV_1_, *∆_WIS_* and *∆_PE_*. † Selected as the best voxel-wise model in the fifth fold. This particular model used the features Jacobian, *ADI*, *SRI* and *PRM­_wos_*

Table S 3 Voxel-wise and aggregate logistic regression models of pCR, features added to the baseline model for each training set and tested on each validation set to of their performances

| **# fold** | Best voxel-wise features | Best aggregate features |
| --- | --- | --- |
| *1^st^* | $Jacobian, ADI, SRI$*,* ${PRM}_{WOS}$ | $\Delta_{SER}$,$\Delta_{WIS}$ |
| *2^nd^* | $Jacobian, ADI, SRI$, ${PRM}_{PE}$ | ${{FTV}_{2}}/{{FTV}_{1}}$, $\Delta_{WIS}$ |
| *3^rd^* | $Jacobian, ADI, SRI$ | ${{FTV}_{2}}/{{FTV}_{1}}$,$\Delta_{WIS}$ |
| *4^th^* | $Jacobian, ADI, SRI$ | ${{FTV}_{2}}/{{FTV}_{1}}$,$\Delta_{PE}$ |
| *5^th^* | $Jacobian, ADI, SRI$, ${PRM}_{WOS}$ | ${{FTV}_{2}}/{{FTV}_{1}}$, $\Delta_{WIS}$,$\Delta_{PE}$ |
| Mean cross-validated AUC (SE) | 0.78(0.03) | 0.71(0.02) |

Table S 4 An example of choosing the best model on a particular fold (fifth fold in this case), from among the best-subset models for 1–7 (voxel-wise) or 1-5 (aggregate) features. All features evaluated as additions to the baseline features. The full model-building procedure does this within each of the five folds.

| **Number of added features** | **Voxel-wise features** | | **Aggregate features** | |
| --- | --- | --- | --- | --- |
|  | Cross-validated C-statistics  (95% CI) | AIC* | Cross-validated C-statistics  (95% CI) | AIC* |
| 1 | 0.75(0.67-0.83) | 270 | 0.69(0.60-0.78) | 276 |
| 2 | 0.77(0.69-0.85) | 266 | 0.69(0.60-0.79) | 275 |
| 3^±^ | 0.78(0.70-0.86) | 263 | **0.70(0.61-0.79)** | **274** |
| 4^†^ | **0.79(0.72-0.87)** | **260** | 0.70(0.61-0.79) | 276 |
| 5 | 0.80(0.72-0.88) | 261 | 0.71(0.62-0.80) | 279 |
| 6 | 0.78(0.71-0.86) | 262 | -- | -- |
| 7 | 0.78(0.70-0.86) | 263 | -- | -- |

*** Lower *AIC* indicates better model, ± Selected subset for aggregate analysis including FTV_2_/FTV_1_ and ∆_SER,_ and ∆_PE_ † Selected subset for voxel-wise analysis including Jacobian, *SRI*, *PRM_PE_* and *PRM_WIS_*

Table S 5 Voxel-wise and aggregate Cox proportional hazard models of RFS, features added to the baseline model for each training set, and the mean of their performances as tested on each validation set.

| **# fold** | Best voxel-wise features | Best aggregate features |
| --- | --- | --- |
| *1^st^* | ${PRM}_{PE}$,${PRM}_{WIS}$, *Jacobian* | ${{FTV}_{2}}/{{FTV}_{1}}$,$\Delta_{WOS}$,$\Delta_{WIS}$ |
| *2^nd^* | ${PRM}_{PE}$, ${PRM}_{WIS}$, *Jacobian, SRI* | $\Delta_{PE}$, $\Delta_{SER}$ |
| *3^rd^* | ${PRM}_{PE}$, ${PRM}_{WIS}$, *SRI* | ${{FTV}_{2}}/{{FTV}_{1}}$,$\Delta_{PE}$ |
| *4^th^* | ${PRM}_{PE}$, ${PRM}_{WIS}$, *Jacobian, SRI* | ${{FTV}_{2}}/{{FTV}_{1}}$,$\Delta_{WOS}$ |
| *5^th^* | ${PRM}_{PE}$, ${PRM}_{WIS}$, *Jacobian, SRI* | $\Delta_{WIS}{, \Delta}_{WOS}$,$\Delta_{SER}$ |
| Mean C-statistic (SE) | 0.76(0.05) | 0.61(0.04) |

Table S 6 Relationship between the proposed imaging features and prediction of outcomes.

| Proposed imaging features | Association of feature values with the outcomes | | |
| --- | --- | --- | --- |
| *ADI* | Higher entropy in orientational deformation | *🡪* | Lower probability of pCR |
| *SRI* | Higher entropy in orientational deformation | *🡪* | Lower probability of pCR and shorter survival |
| *Jacobian* | Higher local volume decrease after NAC | *🡪* | Higher probability of pCR but shorter survival |
| *PRM_PE_* | Higher increase in peak enhancement after NAC | *🡪* | Shorter survival |
| *PRM_WIS_* | Faster enhancement after NAC | *🡪* | Shorter survival |

| **a** | **b** | **c** |
| --- | --- | --- |
| 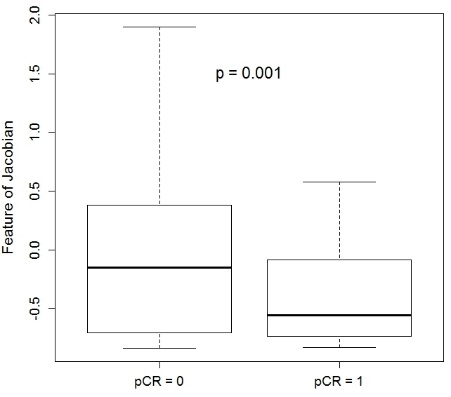 | 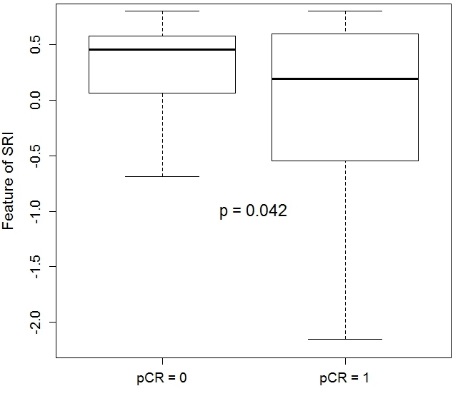 | 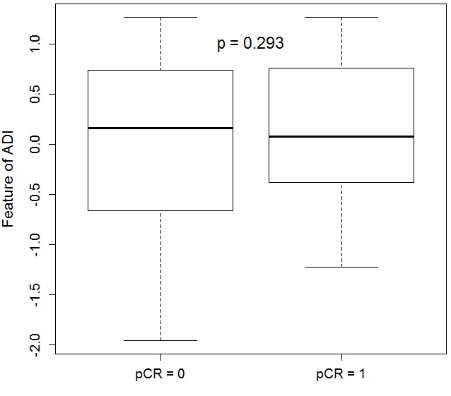 |

Figure S 1 Boxplots showing the distribution of proposed feature values for pCR analysis. *Jacobian* and *SRI* values showed a distinct distribution between two groups of outcomes (*p* < *0.05*) while *ADI* did not indicate distinct distribution.

| **a** | **b** |
| --- | --- |
| 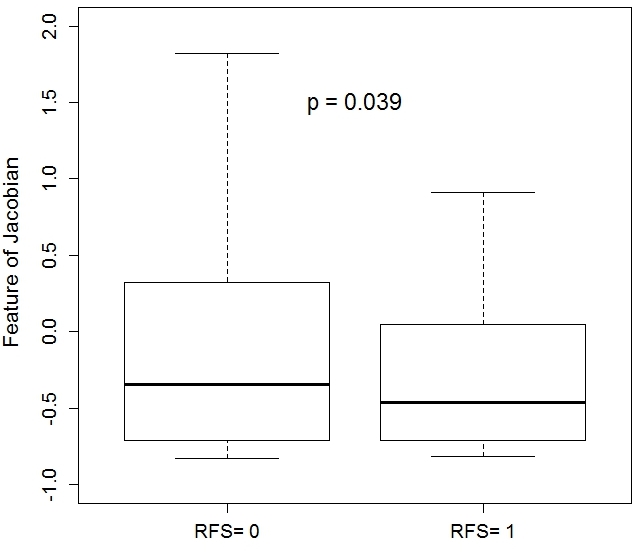 | 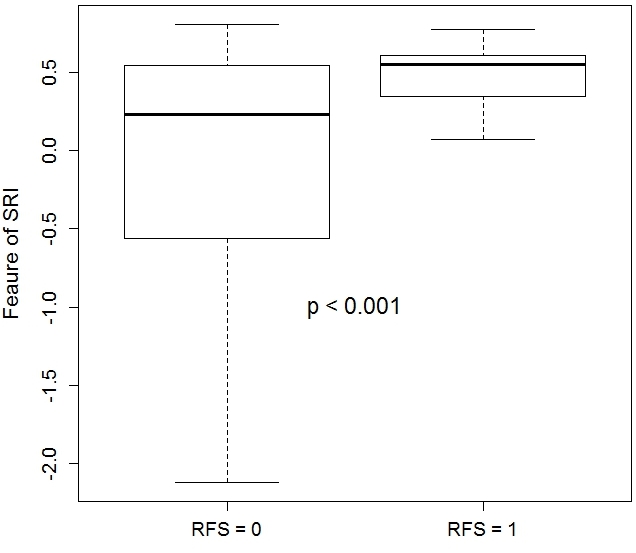 |
| **c** | **d** |
| 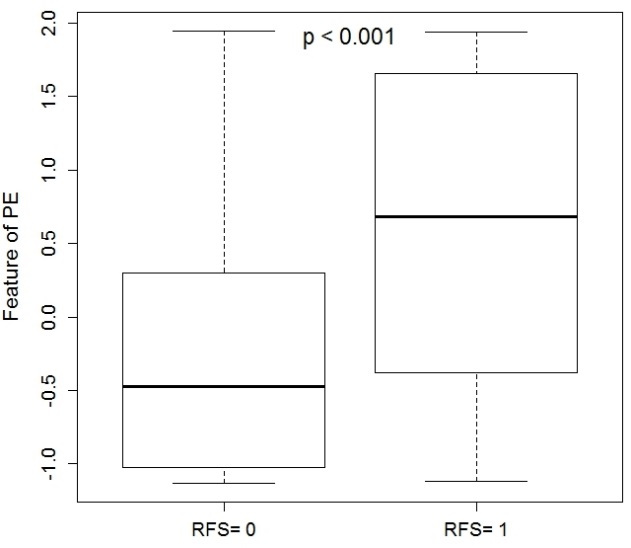 | 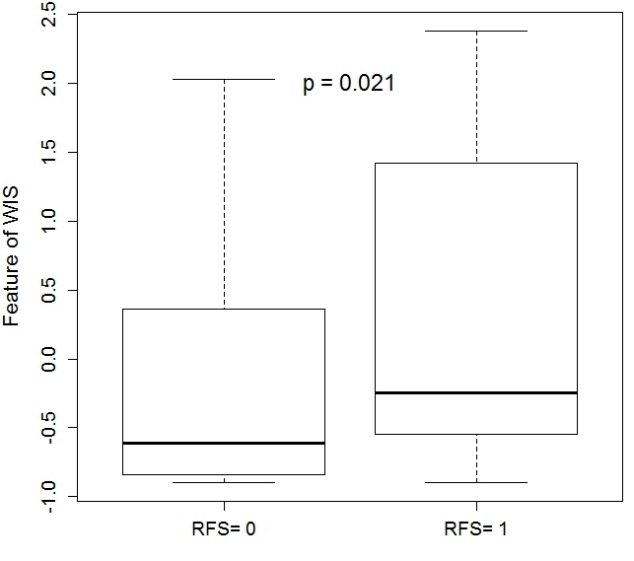 |

Figure S 2 Boxplots showing the distributions of proposed features values for survival analysis. All feature values showed a distinct distribution between two groups of outcomes (*p < 0.05*).
